# Artificial Intelligence and Its Clinical Applications in Orthodontics: A Systematic Review

**DOI:** 10.3390/diagnostics13243677

**Published:** 2023-12-15

**Authors:** Gianna Dipalma, Alessio Danilo Inchingolo, Angelo Michele Inchingolo, Fabio Piras, Vincenzo Carpentiere, Grazia Garofoli, Daniela Azzollini, Merigrazia Campanelli, Gregorio Paduanelli, Andrea Palermo, Francesco Inchingolo

**Affiliations:** 1 Department of Interdisciplinary Medicine, University of Bari “Aldo Moro”, 70124 Bari, Italy; angeloinchingolo@gmail.com (A.M.I.); dott.fabio.piras@gmail.com (F.P.); vincenzo.carpentiere@gmail.com (V.C.); graziagarofoli.g@libero.it (G.G.); daniela.azzollini93@gmail.com (D.A.); merigrazia.92@hotmail.it (M.C.); paduanelli@libero.it (G.P.); francesco.inchingolo@uniba.it (F.I.); 2 Implant Dentistry College of Medicine and Dentistry Birmingham, University of Birmingham, Birmingham B46BN, UK; andrea.palermo2004@libero.it

**Keywords:** artificial intelligence, deep learning, machine learning, orthodontics, diagnosis, treatment planning, treatment monitoring

## Abstract

This review aims to analyze different strategies that make use of artificial intelligence to enhance diagnosis, treatment planning, and monitoring in orthodontics. Orthodontics has seen significant technological advancements with the introduction of digital equipment, including cone beam computed tomography, intraoral scanners, and software coupled to these devices. The use of deep learning in software has sped up image processing processes. Deep learning is an artificial intelligence technology that trains computers to analyze data like the human brain does. Deep learning models are capable of recognizing complex patterns in photos, text, audio, and other data to generate accurate information and predictions. Materials and Methods: Pubmed, Scopus, and Web of Science were used to discover publications from 1 January 2013 to 18 October 2023 that matched our topic. A comparison of various artificial intelligence applications in orthodontics was generated. Results: A final number of 33 studies were included in the review for qualitative analysis. Conclusions: These studies demonstrate the effectiveness of AI in enhancing orthodontic diagnosis, treatment planning, and assessment. A lot of articles emphasize the integration of artificial intelligence into orthodontics and its potential to revolutionize treatment monitoring, evaluation, and patient outcomes.

## 1. Introduction

**Figure 1:**
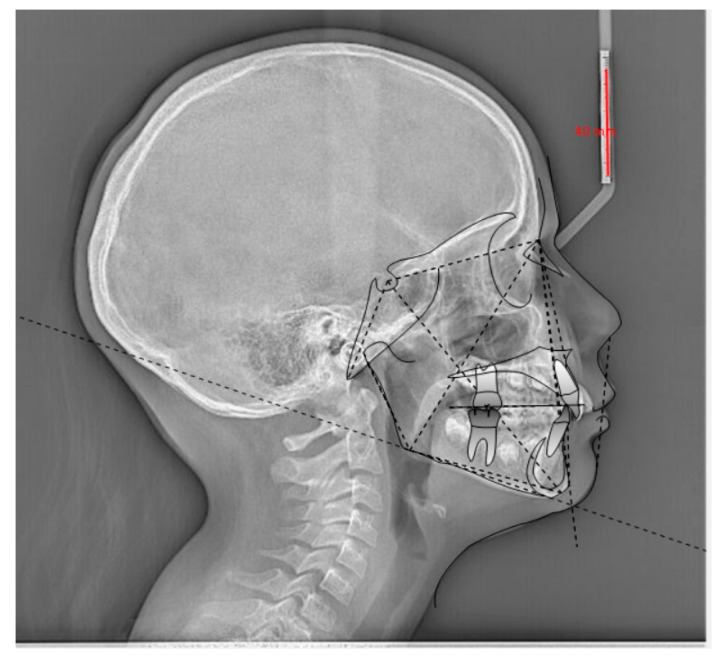
Example of the identification of cephalometric points by AI from a lateral–lateral teleradiographic image required for orthodontic purposes.

Orthodontics has had an incredible development in terms of available technologies with the advent of digital systems: cone beam computed tomography (CBCT), the intraoral scanner, and the software connected to these devices [1]. The integration of deep learning (DL) into software has further accelerated image processing processes [2]. DL is an artificial intelligence (AI) method that teaches computers to process data in a way that is inspired by the human brain. DL models can recognize complex patterns in images, text, sounds, and other data to produce accurate information and predictions [3]. The use of AI by clinicians leads to improved diagnosis, treatment planning, assessment of growth and development, assessment of treatment progress and results, maintenance phase, monitoring at a distance, and long-term follow-up. The term improvement means the possibility of integrating the data collected and selected by the clinician with greater effectiveness and depth [2,4]. As these technological advancements are now widely available, clinicians must be trained and competent in the use of AI-based orthodontic imaging tools. It is essential to know how to orient yourself in the use of AI while considering it a tool to support and not replace the critical and planning skills of humans [5]. One of the orthodontic sectors where the combination of digital images, software, and AI has truly made enormous leaps forward in terms of effectiveness without making the clinician lose his role as the ultimate decision maker is cephalometry, both traditional (2D), based on teleradiograph images in lateral–lateral projection, and 3D cephalometric analysis (CA), developed on 3D rendering models of the skull obtained from scans with CBCT machinery [6,7,8,9,10,11,12,13,14,15,16]. In computer graphics, rendering refers to a process in which special software transforms a two-dimensional image into one that appears more realistic and three-dimensional. This is achieved through precise calculations of perspective, along with the enhancement of the image by adding colors, lights, and shadows, thus giving it depth and a lifelike quality [17]. Analyses have been proposed to identify specific anatomical points and measure different distances, angles, and ratios. For decades, the manual identification of anatomical points was the only option available for cephalometric tracing, but at present, the technological development of AI represents a real and important alternative. Recent software development allows them to collect digital or scanned lateral cephalometric images into their databases to perform automatic identification of cephalometric points (CPs) and CA with tracing and measurements (Figure 1) [18]. This article review attempts to examine several approaches to using artificial intelligence to improve diagnosis, treatment planning, and monitoring in the field of orthodontics.

In terms of positioning of CPs, the use of AI has been evaluated in several studies which, after analyzing 400 to 500 teleradiographs in lateral–lateral projection, have shown an encouraging accuracy in terms of CP positions, ranging between 88.43% and 92%, making the use of AI tools in 3D evaluations of CBCT scans in the field of orthodontics a reality [19,20]. CBCT imaging provides a more complete view of craniofacial structures than traditional 2D X-rays, but manual analysis of CBCT scans can be complex and time-consuming. AI tools have been introduced to simplify and accelerate this process. AI is instrumental in several areas, such as accurately positioning CPs in 3D CA, enabling skeletal diagnoses and growth predictions, and segmenting skeletal structures and teeth. These aspects are crucial in planning maxillofacial surgery [6]. Three-dimensional cephalometry is the analysis of skeletal relationships applied to 3D renderings of the facial mass. To date, there are numerous types of 3D analyses, few of which are accompanied by an exhaustive supply of comparative standard values that allow for not only the linear and angular measurement of bone structures or soft tissues but also the development of a diagnostic meaning of skeletal malocclusion [21]. Here, too, AI has enabled automatic CP positioning and processing skeletal diagnoses in real time. With the rapid advancement of technology, machine learning (ML) (development of specific algorithms) has gained importance in the prediction and classification of pathologies through medical images. This technological expansion in medical imaging has made the automated recognition of anatomical landmarks in radiographs possible, as mentioned above [22]. In this context, ML must be able to not only perform tracings but also develop diagnostic meaning. The potential of ML evolves and grows depending on the data that is made available to the algorithm. AI and ML are used in software dedicated to designing orthognathic cases [23]. AI is used for the segmentation of skeletal tissues and real-time modifications of cephalometric values when designing osteotomy cuts. Even in the field of airway assessment, image processing software has been implemented and speeded up with the help of AI-related technologies. The software can calculate the number of changes in airway volume and plays a key role during the process of planning an orthodontic or orthodontic–surgical treatment [24]. This is because soft tissues exert continuous force due to their inherent flexibility, which could affect the stability of the structural changes achieved. The analysis of airway volume is necessary to determine oral and pharyngeal adaptations to changing respiratory conditions and to evaluate the airway before and after functional orthopedic treatment and orthognathic surgery [25,26]. The use of automated segmentation significantly reduces the time required for airway segmentation and meets the requirements of clinical practice by eliminating the need for manual intervention, which is a laborious and time-consuming process in routine clinical practice and is subject to variations from one operator to another [27]. As regards transparent aligner systems, in addition to highly effective design software, some of these have used DL in treatment plan processing. In this context, DL uses a base of big data that, inserted into algorithm systems, can plan the steps of orthodontic movements necessary for the resolution of a malocclusion [28]. The use of AI can also create a clinical simulation of the patient’s smile after potential orthodontic treatment, creating a previsualization of the result for the patient [29]. This simulation, which takes place using a smartphone app, is an extremely effective tool in emotionally involving the patient in the decision-making process [9]. Some software is equipped with AI techniques that automatically classify permanent (adult) teeth versus primary (baby) teeth and detect the need to compensate for eruption. AI develops automatic segmentation of teeth, starting not only from dicom files but also from .stl files of scans obtained with intraoral scanners, which use ML or DL to predict tooth shapes [2].

## 2. Materials and Methods

### 2.1. Protocol and Registration

PRISMA (Preferred Reporting Items for Systematic Reviews and Meta-Analyses) protocols were followed when conducting this review [30], and it was submitted to PROSPERO (The International Prospective Register of Systematic Reviews) with the number CRD42023475421. 

### 2.2. Search Processing

We searched PubMed, Scopus, Web of Science, and ScienceDirect with a constraint on English-language papers published from 1 January 2013 to 18 October 2023 that matched our topic. The following Boolean keywords were utilized in the search strategy: “artificial intelligence” AND “orthodont*”. These terms were chosen because they best described the goal of our inquiry, which was to learn more about the impact of AI evaluation on the effectiveness of diagnosis, treatment plan, and treatment monitoring.

### 2.3. Inclusion Criteria

All appropriate studies were assessed by three reviewers using the following selected criteria: (1) only studies with human subjects; (2) full text; (3) scientific research evaluating the positive beneficial effects of AI on orthodontic diagnosis, treatment plan, and treatment monitoring. The PICO model was developed in the following manner:

- Population: human subjects;
- Intervention: orthodontic diagnosis, treatment plan, and treatment monitoring;
- Comparison: groups with AI intervention and groups with manual intervention;
- Outcome: diagnosis, treatment plan, and pre- and post-treatment with AI evaluation.

### 2.4. Exclusion Criteria

Exclusion criteria included articles in non-English languages; ineligible research design; ineligible outcome measure; ineligible population; case reports, reviews, and animal studies.

### 2.5. Data Processing

Author differences over article selection were discussed and resolved.

### 2.6. Article Identification Procedure

Two reviewers (F.I. and F.P.) completed the suitability evaluation separately. A manual search was also performed to expand the pool of articles for full-text examination. Articles published in English that fit the inclusion requirements were considered, whilst duplicates and disqualified articles were labeled with the reason for exclusion.

### 2.7. Study Evaluation

The article data were independently evaluated by the reviewers using a special electronic form designed according to the following categories: authors, year of study, type of AI, materials and methods, and results.

### 2.8. Quality Assessment

The quality of the included papers was assessed by two reviewers, R.F. and E.I., using the ROBINS-I tool. ROBINS-I was developed to assess the risk of bias in the results of non-randomized studies that compare the health effects of two or more interventions. Seven points were evaluated and each was assigned a degree of bias. A third reviewer (F.I.) was consulted in the event of disagreement until an agreement was reached. The reviewers were trained in using the ROBINS-I tool and followed the guidelines to evaluate the risk of bias across seven domains: confounding, selection of participants, classification of interventions, deviations from intended interventions, missing data, measurement of outcomes, and selection of reported results. To enhance the objectivity and consistency of the assessments, any discrepancies or disagreements between reviewers were resolved through discussion and consensus. In cases where consensus could not be reached, a third reviewer was involved to make the final determination. Bias assessment using ROBINS-E provided a comprehensive evaluation of potential biases in the non-randomized studies included in this review. It helped to identify the strengths and limitations of the evidence base, contributing to the overall assessment of the quality and reliability of the findings. By considering the risk of bias, this review’s authors were able to make more informed interpretations and draw conclusions based on the available evidence.

## 3. Results

**Figure 2:**
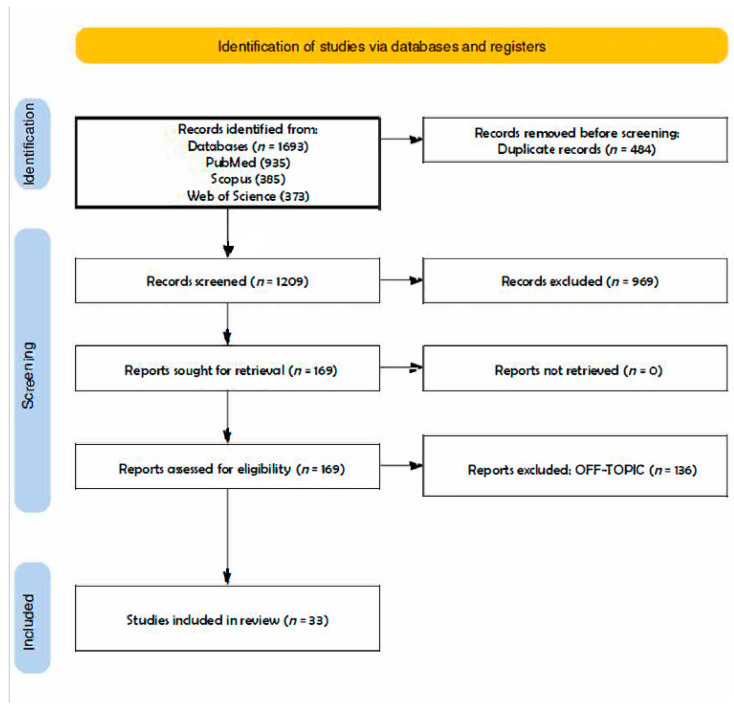
PRISMA (Preferred Reporting Items for Systematic Reviews and Meta-Analyses) flowchart of the literature search and article inclusion process.

A total of 1693 publications were retrieved from the databases PubMed (935), Scopus (385), and Web of Science (373), producing 1209 articles after deleting duplicates (484). The analysis of their titles and abstracts resulted in the exclusion of 969 items. The remaining 169 papers were successfully retrieved and verified for eligibility by the writers. This process resulted in the elimination of 136 items for being off topic. This review comprises the qualitative analysis of the final 33 articles (Figure 2). The items included are schematized in tables at the end of each subsection of the discussion.

## 4. Discussion

### 4.1. Diagnosis

Accurate diagnosis is critical in the field of orthodontics since it is linked to treatment planning and subsequent outcomes. Recently, AI-based diagnosis has been used in treatment planning, which has captured the interest of orthodontists [31]. Ho-Jin Kim et al.’s study aimed to explore the use of a deep convolutional neural network (DCNN)-powered AI model with cephalometric images for categorizing sagittal skeletal relationships [31]. It also aimed to compare the performance of this newly developed DCNN-based AI model to that of an automated tracing AI program. The study included a total of 1574 cephalometric images, which were classified based on the A-point, Nasion (Na), B-point, and ANB angle, with Class I representing 0–4°, Class II > 4°, and Class III < 0° [31]. A test set of 120 images was employed to make comparisons between the AI models. In terms of classifying sagittal skeletal relationships using cephalometric images, the DCNN-based AI model demonstrated superior performance compared to the automated tracing AI software (V-ceph, version 8.3, Osstem, Seoul, Korea) [31]. AI can be also used to evaluate the facial beauty and apparent age of orthognathic patients [32]. The goal of the observational study conducted by R. Patcas et colleagues was to apply AI to describe the influence of orthognathic therapy on facial attractiveness and age appearance [32]. Using specialized convolutional neural networks (CNN) trained on a large dataset of over half a million photos for estimating age and more than 17 million attractiveness ratings, facial attractiveness scores (ranging from 0 to 100) and apparent age were determined for each image [32]. The results were then averaged for each patient, separately for both pre- and post-treatment photographs and in comparison to the actual age (apparent vs. real age) [32]. According to the algorithmic assessments, a significant majority of patients (66.4%) showed improvements in their appearance after treatment, resulting in an average perceived age that was nearly one year younger [32]. AI can also guide the clinician in choosing the most appropriate treatment plan for the patient; Ye-Hyun Kim et al.’s study aimed to explore the use of CNNs for the diagnosis of orthognathic surgery [33]. The research involved 640 patients needing non-surgical orthodontic treatment and 320 requiring surgical treatment. Among these, 150 patients were designated as a test set, and the remaining 810 patients were divided into five groups for fivefold cross-validation. CNN models, including ResNet-18, 34, 50, and 101, were used and evaluated for accuracy, sensitivity, and specificity [33]. In the test set, ResNet-18 performed the best, with an average success rate of 93.80%, followed by ResNet-34 at 93.60%, ResNet-50 at 91.13%, and ResNet-101 at 91.33% [33]. The study provides insights into the ideal characteristics of an AI model’s structure for medical image-based decision-making [33]. Skeletal maturity is vital in determining when and how to proceed with orthodontic treatment. In 2023, Harim Kim and colleagues developed and tested an automated system for evaluating skeletal maturity indicators (SMI) in orthodontics [34]. This system incorporates AI to assess SMI, improving upon existing methods like Greulich and Pyle and Tanner–Whitehouse [34]. It involves three main steps: automated region of interest (ROI) detection, automated SMI evaluation for each region, and mapping SMI stages [34]. The results demonstrate the system’s clinical reliability and its potential to enhance the efficiency and consistency of SMI prediction in clinical practice [34]. Craniofacial development is frequently characterized in terms of magnitude, direction, and velocity [35]. The mandible is the skeletal component with the greatest potential for expansion within the craniofacial complex [35]. ML techniques analyze longitudinal craniofacial cephalometric input data in predicting male postpubertal mandibular length and the Y axis of growth [35]. Tyler Wood et al. employed ML approaches to those using cephalometric data from 163 individuals with Class I Angle malocclusion in their second research study [35]. According to the findings of their study, all of the ML algorithms examined predicted it accurately [35]. The characteristics of the studies are listed in the table below (Table 1).

#### 4.1.1. CA and AI

Identifying anatomic landmarks from a lateral cephalogram is critical for accurate CA to analyze patient growth and occlusion. The use of AI enables the evaluation of lateral radiographs [36].

Bulatova et al. compared a senior orthodontist’s CA of 110 radiographs through Dolphin Imaging^®^ and CA of the same radiographs using AI software Ceppro DDH Inc. Sella (Seoul, Korea) was used as reference landmark to extract x- and y-coordinates for 16 CPs: Nasion (Na), A point, B point, Menton (Me), Gonion (Go), Upper incisor tip, Lower incisor tip, Upper incisor apex, Lower incisor apex, Anterior Nasal Spine (ANS), Posterior Nasal Spine (PNS), Pogonion (Pg), Pterigomaxillary fissure point (Pt), Basion (Ba), Articulare (Art) and Orbitale (Or). There was no statistical difference between CLs analyzed manually by the expert and by AI [37]. 

Two experts manually identified 13 cephalometric landmarks (CLs) that were used as indicators for their detection by a proposed DL model. It analyzed 950 lateral cephalometric images and it was able to perform a fully automatic identification of CLs with an average radial error between the landmarks assigned by one expert and those assigned by the proposed model of 1.84 mm, thus achieving excellent results [38].

Sixty-six CLs and ten linear and angular measurements featured in Arnett’s analysis were considered by an expert and by CEFBOT (RadioMemory Ltd., Belo Horizonte, Brazil) on thirty radiographs. CLs and measures were replicated with a 15-day delay between measurements for both procedures, and there was no statistically significant difference in results [39].

An automated CA based on a customized DL algorithm was comparable to measurements made by 12 examiners [5].

Kim et al. had 3150 lateral cephalograms analyzed by a DL model taking into account the gold standard values of CLs obtained by two orthodontists on 100 lateral cephalograms using the V-ceph software((V-ceph, version 8.3, Osstem, Seoul, Korea). It was noted that its accuracy of CL recognition was comparable with that of two orthodontists with more than 10 years of clinical experience [40]. 

Jeon et al. found that automatic CA performed using a CNN had admissible diagnostic results but it needed careful attention and additional manual control to achieve higher accuracy [41]. Another study found AI in combination with manual analysis to be useful [42].

Gökhan Çoban et al. compared the values of CLs obtained by cephalometry using Dolphin^®^ v. 11.5, Chatsworth, CA, USA, and those obtained using the WebCeph platform (AI). Differences were detected in some CL measurements [43]. Ioannis A Tsolakis et al. compared the values of CLs obtained by cephalometry using the semi-automated software Dolphin 3D Imaging program^®^ version 11.0 and those obtained using CS imaging V8 software (AI). They found that all cephalometric measurements were accurate [44]. Britta Ristau et al. compared the values of CLs obtained by cephalometry using AudaxCeph^®^’s automatic tracing software (version 6.3.11.4346) and those obtained by a semi-automated approach by human examiners using the same software [45]. There was no statistical difference between the results obtained by the orthodontists and AudaxCeph^®^’s automatic tracing software, except for Porion and L1 apex [46]. 

In another study, three methods were used to execute cephalometric measurements: WebCeph software (AssembleCircle Corp., Gyeonggi-do, Republic of Korea), WebCeph software after manual modification of LM, and manual CL detection and digital measurement generation using OnyxCeph software (https://onyxceph.eu/en/programmversionen/ accessed on 14 November 2023) AI followed by hand CL adjustment might be an accurate strategy in CA [47].

In yet another study, three methods were used to execute cephalometric measurements: Dolphin Imaging 13.01^®^ (Dolphin Imaging and Management Solutions, Chatsworth, CA, USA), app-aided tracing using the CephNinja 3.51 app, and fully automated web-based tracing with CephX [48]. CephX analysis with manual correction had the potential to be employed in clinical practice because it was equivalent to CephNinja and Dolphin^®^ and required little time [49]. The characteristics of the studies are listed in the table below (Table 2).

#### 4.1.2. AI-Guided Assessment of Vertebral Maturation

The assessment of skeletal maturity is very important in treatment planning for optimal treatment timing. A custom-design CNN model consisting of two parts, feature extraction and classification, was used to classify CVM into six maturation stages (CS1–CS6) [50,51]. AggregateNet was utilized in the model for feature extraction, and as the preprocessing layer, it used directional filters to enrich the information. The AggregateNet output feature was coupled with age input to produce conclusions as well as to boost network performance [51]. Hatice Kök et al. [52] used seven algorithms to determine CVS: k-nearest neighbors (k-NN), naive Bayes (NB), decision tree (DT), artificial neural network (ANN), support vector machine (SVM), random forest (RF), and logistic regression (LR). These algorithms showed low and high values for each stage. The ANN algorithm was a steadier algorithm than others in determining cervical vertebrae stages [52]. In 2021, Seo et al. assessed and compared the performance of six cutting-edge CNN-based DL models for cervical vertebral maturation (CVM) using lateral cephalometric radiographs [53]. A dataset of lateral cephalometric radiographs was collected from patients aged 6–19 years, and six pretrained CNN architectures were used for CVM stage classification. Data augmentation techniques were applied to mitigate overfitting, and model performance was evaluated using accuracy, recall, and precision. The models successfully achieved over 90% accuracy in classifying CVM stages. Grad-CAM visualization revealed that different models focused on distinct areas of the cervical vertebrae for classification. Overall, this study demonstrated the potential of DL models using lateral cephalometric radiographs in aiding clinicians in accurate diagnosis and treatment planning related to skeletal maturity [53]. Seo et al. [54] delved into the critical area of bone age assessment, an essential tool in pediatrics, endocrinology, and orthodontics for evaluating child and adolescent development. Their article provides an overview of a novel approach, known as the “Deep Focus Approach” (DFA), which employs DL techniques to improve the accuracy and efficiency of bone age estimation using lateral cephalograms. Bone age assessment is a vital diagnostic tool used to evaluate an individual’s skeletal development in comparison to chronological age. Accurate assessments are crucial in diagnosing and treating various growth-related conditions, such as growth hormone deficiencies or precocious puberty. Traditionally, radiologists and clinicians have relied on manual assessment methods, which are time-consuming, subject to interobserver variability, and may not always provide precise results. Therefore, the integration of DL techniques for automated bone age estimation is a significant step forward in this field. The DFA appears to offer several notable advantages in bone age estimation. One of its key features is the use of DL algorithms, specifically CNNs. These networks have shown remarkable potential in image recognition tasks and have been successfully applied in medical image analysis [55]. In this context, CNNs are trained to detect and evaluate specific features related to bone development in lateral cephalograms, enabling the system to provide more accurate and consistent assessments. The article suggests that this approach minimizes the subjectivity associated with manual readings, as the DL model follows a standardized methodology [56]. It is critical to note that this method is not intended to replace the expertise of a trained radiologist or clinician but rather to augment their capabilities, improve accuracy, and save time. Additionally, the article discusses the DFA’s potential for scalability and broader applicability. This approach could be integrated into radiology departments and orthodontic practices to streamline the bone age assessment process. It may also be used in telemedicine scenarios, where remote consultations require accurate and consistent assessments. The article’s focus on DL techniques in the context of bone age estimation aligns with the broader trend of leveraging AI in medical imaging and diagnostics [57]. AI has shown great promise in automating repetitive and time-consuming tasks, thus allowing healthcare professionals to focus on more complex and critical aspects of patient care. While the DFA holds considerable promise, it is critical to address certain challenges and limitations. For instance, the quality of lateral cephalograms can vary significantly, and the model’s performance may be affected by artifacts or unusual anatomical features. Moreover, the need for a substantial dataset for training the DL model is an important consideration. The quantity and diversity of images used for training can greatly influence the model’s generalization to new data. In conclusion, it represents a significant advancement in the field of pediatric and orthodontic diagnostics. By harnessing the power of DL, this approach has the potential to enhance the accuracy and efficiency of bone age assessments, benefiting both patients and healthcare professionals. As medical imaging and AI continue to advance, innovative approaches like this will likely play an increasingly important role in improving healthcare delivery and patient outcomes. Further research and validation studies will be crucial to determine the reliability and accuracy of this approach in clinical settings [54]. Studies on AI in orthodontics have been carried out to evaluate the processes of cervical vertebral maturation (CVM). In 2023, Gülsün Akay et al. evaluated 588 lateral cephalometric x-ray images using a DL-based CNN model. Patients ranged in chronological age from 8 to 22 years [58]. The CVM stages in the images were divided into six subgroups based on the bone maturation process. With this study, it was demonstrated that the developed model achieved a classification accuracy of 58.66% in the evaluation of CVM stages [59]. The primary objective of Hakan Amasya’s study was to create five distinct supervised ML classifier models using AI techniques to assess CVM and then compare their performance [60]. Additionally, the researchers developed a clinical decision support system (CDSS) to enhance the objectivity of the results [60]. A custom software was employed to manually label the samples, and an integrated CDSS was created after evaluating 100 radiographs. Each radiograph was marked with 26 points, and the CDSS provided suggestions based on these points and the CVM analysis conducted by a human observer [60]. Subsequently, 54 features for each sample were saved in text format and classified using various models, including LR, SVM, RF, ANN, and DT [60]. The results indicated that the best performance was achieved with the ANN model. For cervical vertebrae morphology classifier models, the LR model yielded the best results for determining the presence of concavity, while the DT model performed well in identifying vertebral body shapes [60]. The characteristics of the studies are listed in the table below (Table 3).

### 4.2. AI-Guided Treatment Plan

AI is proving to be a valuable ally in the design of customized orthodontic treatment plans. Numerous research studies have investigated the use of ML-based clinical decision support models in this context.

One study of particular interest focused on the application of AI and ML in orthodontics to predict diagnoses and treatment plans. This research study involved a large sample of 700 patients undergoing orthodontic treatment, analyzing clinical, cephalometric, and photographic data [61]. The research team developed four separate ML models aimed at identifying jaw bases, suggesting the appropriateness of dental extractions and proposing solutions to correct protruded or retruded jaw bases. The results demonstrated an average accuracy of 84% in predicting treatment plans, highlighting the robustness of these models. Furthermore, the study identified significant correlations between diagnostic parameters and key factors in orthodontic planning [62].

In the same manner, research has been conducted on the issue of variability in decisions regarding extractions in orthodontic treatment. The lack of a standard formula for such decisions, often based on the experience and clinical assessment of orthodontists, has led to divergent results among practitioners, undermining the standardization of the treatment planning process. In response to this problem, a study conducted by Mason employed ML to predict tooth extraction decisions, demonstrating the effectiveness of this approach, especially when using SVM [63]. Similarly, Etemad et al. trained an ANN to assist in orthodontic extraction treatment decisions, achieving significant improvements in the consistency and effectiveness of treatment choices [64]. A further study conducted by Jung exploited ANN for similar purposes, using clinical data, radiographs, and measurements to take into account aspects such as the need for extractions, their type (identical or differential), and the amount of retraction required [65].

In summary, these studies highlight the extraordinary potential of AI in orthodontic treatment planning. AI can reduce variability in practitioners’ decisions and improve the consistency and effectiveness of treatment choices. However, it is crucial to emphasize that AI should be seen as a valuable tool to support human experience, rather than a complete surrogate for orthodontists’ clinical judgment.

In addition, other researchers have aimed at developing predictive models for orthognathic surgery planning in patients with skeletal class III. For example, a study conducted by Lee et al. involved a sample of 196 patients selected based on specific skeletal and orthodontic characteristics, using a wide range of cephalometric and demographic data to train ML models such as random forest (RF) and logistic regression (LR). Both models achieved high accuracy of up to 90% in treatment planning [66]. Furthermore, a study by Chaiprasittikul used an ANN to classify patients according to the need for orthognathic surgery, achieving a remarkable 96.3% diagnostic agreement rate in surgical decisions [67].

Overall, this review highlights the potential of AI as a complementary tool in orthodontic treatment planning, promoting a more evidence-based clinical practice that is targeted to the specific needs of the patients. The characteristics of the studies are listed in the table below (Table 4).

### 4.3. Orthodontic Treatment Monitoring

Caruso et al. [68] discussed the Dental Monitoring System (DMS), a knowledge-based algorithm designed for the automatic monitoring of orthodontic treatment. They provide two examples of how this technology was used. To provide a thorough and automated method of tracking the progress of orthodontic treatment, the DMS makes use of a variety of technologies, including AI [69]. An important innovation in orthodontics is the application of AI since it allows for precise and continual treatment monitoring. To assess data from orthodontic patients and effectively track development, the algorithm makes use of knowledge-based methods. The DMS can improve the precision and efficacy of orthodontic treatment by automating the monitoring procedure [68]. Lee et al. [70] delve into the accuracy of integrated dental models utilizing DL by combining intraoral scans and CBCT scans. Since it permits precise and continual treatment monitoring, the application of AI in orthodontics represents a considerable advancement [71]. To assess data from orthodontic patients and effectively track development, the algorithm makes use of knowledge-based methods [26]. The DMS can improve the precision and efficacy of orthodontic treatment by automating the monitoring procedure [70]. Ferlito et al. [72] investigate the effectiveness of utilizing AI in remote monitoring of clear aligner therapy, a popular orthodontic treatment method [4]. Their paper offers a future assessment, analyzing the advantages and results of remote monitoring powered by AI. A useful technique for remotely monitoring patients receiving clear aligner treatment is AI. It can perform data analysis, monitor development, and spot any treatment plan deviations. By giving orthodontists quick information, this technology enables them to make the necessary modifications remotely [73]. The research demonstrates the probable potential of AI in enhancing patient compliance and treatment effectiveness in clear aligner therapy [72]. Patcas et al. [32] explore the application of AI to assess how orthodontic treatment impacts facial attractiveness and estimated age. Here, orthodontics and aesthetics come together, intriguingly, with AI being crucial in assessing treatment effectiveness and analyzing face traits. The results of orthodontic therapy may be objectively measured in terms of changes to the appearance of the face [74]. Offering useful insights for both practitioners and patients, it offers a systematic method for assessing the influence of therapy on attractiveness and apparent age [20]. This use of AI brings a fresh perspective to the evaluation of orthodontic treatment, emphasizing not just functional advancements but also the improvement of overall face attractiveness [32]. The characteristics of the studies are listed in the table below (Table 5).

### 4.4. Quality Assessment and Risk of Bias

**Figure 3:**
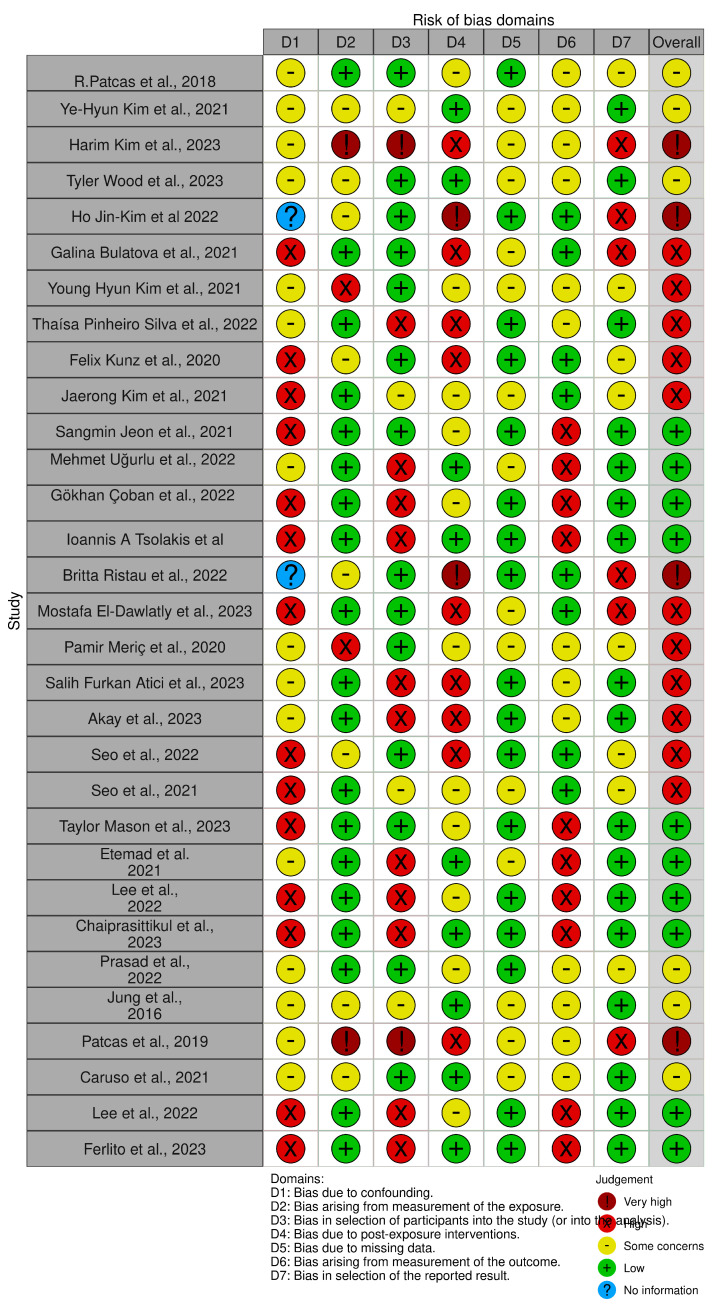
Quality assessment of bias [5,31,32,33,34,35,37,38,39,40,41,42,43,44,46,47,49,51,53,54,59,62,63,64,65,66,67,68,70,72].

The risk of bias in the included studies is reported in Figure 3. Regarding bias due to confounding, most studies have a high risk. Bias arising from measurement is a parameter with a low risk of bias. The majority of studies have a low risk of bias due to participant selection bias. Bias due to postexposure cannot be calculated due to high heterogeneity. Bias due to missing data is low in the majority of studies. Bias arising from the measurement of outcomes is low. Bias in the selection of the reported results is high in the majority of studies. The final results show that ten studies have a low risk of bias, ten studies have a high risk of bias, four have a very high risk of bias, and the remainder have a questionable risk of bias.

## 5. Conclusions

AI has revolutionized the orthodontic field and continues to improve it through multiple applications, from analyzing skeletal relationships to assessing facial attractiveness and predicting postpuberty mandibular growth. This review has shown that numerous experiments evaluate the validity of AI in the diagnosis phase, in treatment planning, and in the choice of the most appropriate therapy for the patient, achieving results comparable to those obtained manually by experts. Integrating AI into orthodontic assessments enhances treatment monitoring, especially with algorithms such as DMS, which provide precise automatic analysis. The possibility of integrating intraoral scans and CBCT offers new perspectives for the 3D evaluation of root position, which is useful for predicting the displacement of the entire tooth and not just the dental crown. The DMS and similar algorithms showcase the power of knowledge-based approaches, DL, and AI in providing automated and precise monitoring of orthodontic treatment progress. Furthermore, the application of AI in remote monitoring and assessment of clear aligner therapy demonstrates how technology can enhance treatment efficiency and patient compliance. The capacity of AI to objectively measure the influence of orthodontic treatment on facial appearance and predicted age gives a more comprehensive view of treatment outcomes. Overall, these publications illustrate the exciting advances that AI offers in the area of orthodontics, which have the potential to change the way orthodontic treatments are monitored, assessed, and tailored. Despite the immense potential offered by AI, it remains crucial to emphasize that the execution of orthodontic therapy remains firmly in the hands of the clinical expert. These innovations, although revolutionary, are complementary tools that assist and enrich the work of orthodontic professionals, contributing to a more precise and personalized approach to patient care.

## Figures and Tables

**Table 1:** Characteristics of the studies.

Authors/Years	Type of Study	Type of AI	Materials and Methods	Results
R. Patcas et al., 2018 [32]	Observational study	ANN	Photographs of consecutive orthognathic patients were taken before and after treatment.	According to the algorithmic assessments, a significant majority of patients (66.4%) showed improvements in their appearance after treatment, resulting in an average perceived age that was nearly one year younger.
Ye-Hyun Kim et al., 2021 [33]	Observational study	ANN (ResNet-18, ResNet-34, ResNet-50 and ResNet-101)	The study included individuals who needed non-surgical orthodontic therapy and surgical orthodontic treatment.	ResNet-18 is the best model for orthognathic surgery diagnosis, providing important insights into the ideal characteristics of an AI framework for medical image-based decision-making.
Harim Kim et al., 2023 [34]	Observational study	AI-based automated assessment system	The dataset used for primary verification of the AI-based automated assessment system for Fishman’s SMI consisted of hand–wrist radiographs.	AI-based automated assessment system has proven to provide highly accurate SMI prediction with minimal errors.
Tyler Wood et al., 2023 [35]	Retrospective study	ML	Cephalometric data with Class I Angle malocclusion were utilized to train several ML methods. ANOVA was used to analyze the differences.	All of the ML systems tested properly predicted postpubertal mandibular length and Y axis of growth.
Ho Jin-Kim et al., 2022 [31]	Retrospective study	DCNN	A total of 1574 cephalometric pictures were included in the study.	The micro-average values of the DCNN-based AI model surpassed the automated tracing AI program in terms of performance.

**Table 2:** Characteristics of the studies.

Authors/Years	Type of Study	Type of AI	Materials and Methods	Results
Galina Bulatova et al., 2021 [37]	Retrospective study	AI software Ceppro DDH Inc. (Seoul, Korea)	Lateral cephalograms were analyzed by a calibrated senior orthodontic resident using Dolphin Imaging^®^ and the same images were uploaded to the AI software Ceppro DDH.	There was no statistical difference in manually analyzed CLs and those obtained by AI.
Young Hyun Kim et al., 2021 [38]	Retrospective study	The developed DL model has a two-step structure.	Two examiners manually identified the 13 most important CLs to set as references. The landmarks were automatically measured using the proposed model in lateral cephalometric images.	The proposed DL model can perform fully automatic identification of CLs.
Thaísa Pinheiro Silva et al., 2022 [39]	Retrospective study	CEFBOT (RadioMemory Ltd., Belo Horizonte, Brazil)	An expert and CEFBOT evaluated the 66 landmarks and 10 linear and angular measures featured in Arnett’s analysis on the radiograph.	CEFBOT (https://www.radiomemoryglobal.com/#h.r8d6r24868b accessed on 14 November 2023) software can be considered a promising tool.
Felix Kunz et al., 2020 [5]	Retrospective study	A customized open-source CNN DL algorithm (Keras and Google TensorFlow) is directed toward analyzing visual imagery and has an input layer, multiple hidden layers, and an output layer.	Both AI and each examiner analyzed 12 orthodontic parameters based on cephalometric images.	No clinically relevant difference was noticed between the two analyses.
Jaerong Kim et al., 2021 [40]	Retrospective study	A cascade network consisting of ROI detection and landmark prediction.	Two orthodontists evaluated 100 lateral cephalograms and the mean of these values was considered the gold standard. The DL model evaluated 3150 lateral cephalograms.	The overall automated detection error was 1.36 ± 0.98. The accuracy of CL recognition was comparable with that made by two orthodontists with more than 10 years of clinical experience.
Sangmin Jeon et al., 2021 [41]	Retrospective study	CephX for the AI analysis.	The cephalograms were analyzed with V-ceph for the conventional CA and with CephX for the AI analysis.	Variations were found in saddle angle, linear measurements of maxillary incisor to NA line, and mandibular incisor to NB line.
Mehmet Uğurlu et al., 2022 [42]	Retrospective study	AI system (CranioCatch, Eskisehir, Turkey).	A CNN-based AI algorithm for automatic CL detection was developed and used to detect CLs.Then, an orthodontist with 9 years of experience analyzed the CA of the AI.	There were no statistical differences between manual identification and AI groups in 11 out of 16 points. AI increased the efficiency of CL identification.
Gökhan Çoban et al., 2022 [43]	Retrospective study	WebCeph was used for AI-based CA.	Differences between using the semi-automated software Dolphin^®^ (v. 11.5, Chatsworth, CA, USA) and WebCeph (WEBCEPH™, Artificial Intelligence Orthodontic & Orthognathic Cloud Platform, South Korea, 2020) software for each CL.	It was determined that there was a noticeable change between SNB, ANB, and SN.PP, U1.SN, U1-NA, U1.NA, L1-APog, IMPA, L1-NB, and ULE.
Ioannis A Tsolakis et al. [44]	Retrospective study	CS imaging V8 software was used for AI-based CA.	The difference between using semi-automated software Dolphin^®^ 3D Imaging program (version 11.0) and CS imaging V8 software for each CL.	There were no significant differences between the two methods (*p* > 0.0027) for the SN-MP, U1-SN, SNA, SNB, ANB, L1-NB, SNPg, ANPg, SN/ANS-PNS, SN/GoGn, U1/ANS-PNS, L1-APg, U1-NA, and L1-GoGn landmarks.
Britta Ristau et al., 2022 [46]	Retrospective study	AudaxCeph^®^’s automatic tracing software.	The difference between AudaxCeph^®^’s automatic tracing and a semi-automated approach by human examiners using the same software.	AudaxCeph^®^ was a reliable resource for clinicians in analyzing orthodontic cases, even if there were unreliable points, such as Porion, Orbitale, U1 apex, and L1 apex.
Mostafa El-Dawlatly et al., 2023 [47]	Retrospective study	WebCeph software and OnyxCeph software.	Lateral cephalometric radiographs were evaluated.	Fewer differences were obtained with the modified WebCeph software method than with the OnyxCeph method.
Pamir Meriç et al., 2020 [49]	Retrospective study	Dolphin Imaging^®^ 13.01, app-aided tracing using the CephNinja 3.51 app, and fully automated web-based tracing with CephX.	Three methods were used to execute cephalometric measurements: Dolphin Imaging^®^ 13.01, app-aided tracing using the CephNinja 3.51 app, and fully automated web-based tracing with CephX.	Manual correction of CephX landmarks gave similar outcomes to digital tracings using CephNinja and Dolphin^®^.

**Table 3:** Characteristics of the studies.

Authors/Years	Type of Study	Type of AI	Materials and Methods	Results
Salih Furkan Atici et al., 2023 [51]	Retrospective study	A DL network is shown, and a parallel structured DCNN with a preprocessing layer that uses X-ray pictures and age as input is proposed.	A custom CNN model with two sections, feature extraction and classification, was employed to categorize CVM into six maturation phases (CS1–CS6). AggregateNet was utilized in the model for feature extraction, while directional filters were employed as the preprocessing layer to improve the information.	AggregateNet, when combined with adjustable directional edge filters, outperformed other models with fully automated CVM stage determination.
Akay et al., 2023 [59]	Retrospective study	DL-based CNN	Digital lateral cephalometric radiographs of patients between 8 and 22 years were evaluated.	The study demonstrated that the developed model achieved moderate success.
Seo et al., 2022 [54]	Retrospective study	DeepLabv3, a semantic segmentation network for delimited cervical vertebral region, and Inception-ResNet-v2, a classification network converted to a regression model for age estimate, were used.	The study included 900 people between the ages of 4 and 18 who had a lateral cephalogram and a hand–wrist radiograph on the same day. First, the cervical vertebrae were segmented from the lateral cephalogram using DeepLabv3 architecture. Second, after isolating the region of interest from the segmented picture for preprocessing, bone age was estimated using transfer learning and an Inception-ResNet-v2 architecture-based regression model.	Using the gradient-weighted regression activation map methodology, key regions were visualized on cervical vertebral imaging to create a prediction.
Seo et al., 2021 [54]	Retrospective observational study	CNN	600 lateral cephalometric radiographs of patients aged 6–19 years; CNNs were used for CVM classification.	Achieved more than 90% accuracy in classifying CVM phases.

**Table 4:** Characteristics of the studies.

Authors/Years	Type of Study	Type of AI	Materials and Methods	Results
Taylor Mason et al., 2023 [63]	Retrospective observational study	ML (LR, RF, SVMs, ANN)	393 patients, a diverse population. Trained LR, RF, SVM, and ANN on 70% of data, and tested on 30%. Evaluated accuracy and precision for extraction decisions.	High accuracy in predicting tooth extraction decisions.
Etemad et al., 2021 [64]	Retrospective observational study	ANN, RF	838 orthodontic patient records. Split into extraction and non-extraction samples. Used 117 clinical and cephalometric variables for ML (RF and MLP) for tooth extraction prediction.	High accuracy in predicting tooth extraction therapy.
Lee et al., 2022 [66]	Retrospective observational study	ML (RF, LR)	196 skeletal class III patients, 136 training, 60 tests. Estimated neural network success rate. Binary classifier for surgical case prediction.	AI is useful for successfully classifying patients up to 90% of candidates for surgery.
Chaiprasittikul et al., 2023 [67]	Retrospective observational study	ANN	Analysis of 538 cephalometric radiographs using Detectron2 and ANN. Developed neural network decision support system for orthognathic surgery prediction.	AI is useful for successfully classifying up to 90% of candidates for surgery.
Prasad et al., 2022 [62]	Retrospective observational study	ML (extreme gradient boosting, RF, decision tree)	Analyzed 700 orthodontic cases with 33 inputs and 11 outputs. Developed ML models and compared their predictions with expert orthodontist decisions.	The overall accuracy of the models was 84%.
Jung et al., 2016 [65]	Retrospective observational study	ANN	Analyzed 156 patients with 12 cephalometric variables, 6 indexes, and 3-bit extraction pattern diagnosis. Created and evaluated ANN.	Effectiveness in assisting professionals in decision-making with success rates of 84–93%.

**Table 5:** Characteristics of the studies.

Authors Years	Type of Study	Type of AI	Materials and Methods	Results
Patcas et al., 2019 [32]	Observational study	AI to explain how orthodontic therapy affects facial beauty and apparent age.	Every photograph has patient-related information (patient age, sex, malocclusion, and surgeries performed) tagged on it. With specialized CNNs trained on >0.5 million photos for age estimation and with >17 million attractiveness ratings, face attractiveness (score: 0–100) and apparent age were determined for each image.	The algorithms discovered that the vast majority of patients’ looks improved following therapy (66.4%), leading to a roughly one-year younger appearance, especially after profile-altering surgery. Similar positive effects of orthognathic therapy on beauty were seen in 74.7% of cases, particularly following lower jaw surgery.
Caruso et al., 2021 [68]	Case report	Correct biomechanics were guaranteed by the software’s analysis of the aligner’s fit and retention.	Depending on the chosen protocol, guided scanning will transmit 20–30 photos to the servers for processing, which may be broken down into four steps: Step 1: The system processes the raw photos. They are evaluated for quality to see if the patient requires another scan or not; Step 2: Using a prediction score (% of certainty), the algorithm can locate teeth and identify them. In some orthodontic extraction situations, the technology is so sophisticated that it can sometimes tell if a tooth is a first or second premolar. Additionally, the gingiva is shown; Step 3: Finding the various clinical parameters; Step 4: The AI will review the data and, using the selected strategy, will provide instructions to the patient and the team.	The patient demonstrated exceptional compliance with and confidence in the DM system on receiving nearly all “GO” signals during his therapy. Up to the completion of all clinical objectives, monitoring was activated. Therefore, it was just put on hold while awaiting the new aligners.
Lee et al., 2022 [70]	Clinical Study	To compare the creation of integrated tooth models (ITMs) with the manual method and to assess the accuracy of DL-based ITMs by combining intraoral scans and CBCT scans for three-dimensional (3D) root position evaluation during orthodontic treatment.	15 patients who underwent orthodontic treatment with premolar extraction had intraoral scans and related CBCT scans taken before and after treatment.	The procedure times taken to obtain the measurements were longer in the manual method than in the DL method.
Ferlito et al., 2023 [72]	Prospective study	Clear aligner treatment has lately gained popularity due to the use of AI for remote monitoring. DL algorithms on a patient’s mobile smartphone were used to decide readiness to move to the next aligner (i.e., “GO” versus “NO-GO”) and detect places where the teeth do not match the clear aligners.	Thirty patients under treatment with clear aligners at an academic clinic were scanned twice using a remote smartphone monitoring software, and the results were compared.	A 44.7% gauge compatibility was observed. Between Scan 1 and 2, 83.3% of patient instructions agreed; however, 0% agreed on whether and/or how many teeth had tracking difficulties. In the mesiodistal, buccolingual, occlusogingival, tip, torque, and rotational dimensions, patients who received a “GO” instruction exhibited mean differences of 1.997 mm, 1.901 mm, 0.530 mm, 8.911, 7.827, and 7.049, respectively. These differences were not statistically significant when patients were given “NO-GO” instructions.

## Data Availability

Not applicable.

## Abbreviations

AI	Artificial Intelligence
ANB	Angle between point A, point B, and nasion
ANS	Anterior Nasal Spine
Art	Article
Ba	Basion
CA	Cephalometric Analysis
CBCT	Cone Beam Computed Tomography
CDSS	Clinical Decision Support System
CL	Cephalometric Landmark
CNN	Convolutional Neural Network
CVM	Cervical vertebral maturation
DCNN	Deep Convolutional Neural Network
DFA	Deep Focus Approach
DG	Digital Manual
DL	Deep Learning
DT	Decision Tree
Go	Gonion
ICC	Intraclass Correlation Coefficient
ITMs	Integrated Tooth Models
k-NN	k-Nearest Neighbors
LR	Logistic Regression
Me	Menton
ML	Machine Learning
Na	Nasion
NB	Naive Bayes
Or	Orbitale
PNS	Posterior Nasal Spine
Pg	Pogonion
PRISMA	Preferred Reporting Items for Systematic Reviews and Meta-Analyses
PROSPERO	The International Prospective Register of Systematic Reviews
Pt	Pterigomaxillary fissure point
RF	Random Forest
ROI	Region of Interest
SMV	Skeletal Maturity Indicators
SVM	Support Vector Machines
